# Bioactive Compounds in *Salicornia patula* Duval-Jouve: A Mediterranean Edible Euhalophyte

**DOI:** 10.3390/foods10020410

**Published:** 2021-02-12

**Authors:** Irene Sánchez-Gavilán, Esteban Ramírez, Vicenta de la Fuente

**Affiliations:** Departamento de Biología, Facultad de Ciencias, Universidad Autónoma de Madrid, Cantoblanco, 28049 Madrid, Spain; irene.sanchezgavilan@estudiante.uam.es (I.S.-G.); esteban.ramirez@uam.es (E.R.)

**Keywords:** halophytes, phenolics, flavonoids, fatty acids, Tinto river, characterization bioactive compounds

## Abstract

Many halophytes have great nutritional and functional potential, providing chemical compounds with biological properties. *Salicornia patula* Duval-Jouve is a common euhalophyte from saline Mediterranean territories (Spain, Portugal, France, and Italy). In the present work we quantified for the first time the bioactive compounds in *S. patula* (total phenolic compounds and fatty acids), from Iberian Peninsula localities: littoral-coastal Tinto River basin areas (southwest Spain, the Huelva province), and mainland continental territories (northwest and central Spain, the Valladolid and Madrid provinces). Five phenolic acids including caffeic, coumaric, veratric, salicylic, and transcinnamic have been found with differences between mainland and coastal saltmarshes. *S. patula* contain four flavonoids: quercetin-3-O-rutinoside, kaempferol/luteolin, apigenin 7-glucoside, and pelargonidin-3-O-rutinoside. These last two glycosylated compounds are described for the first time in this genus of Chenopodiaceae. The fatty acid profile described in *S. patula* stems contains palmitic, oleic, and linoleic acids in high concentrations, while stearic and long-chain fatty acids were detected in low amounts. These new findings confirm that *S. patula* is a valuable source of bioactive compounds from Mediterranean area.

## 1. Introduction

Species in the Chenopodiaceae family have been proven to have a high content of minerals, polyphenols, fatty acids, and other compounds. The presence of bioactive compounds in this type of plant represents a challenge due to its possible uses both at a culinary and industrial level [1]. *Salicornia* species (Chenopodiaceae, Salicornioideae) respond to saline stress by making osmotic adjustments and anatomical, physiological, and metabolic adaptions, and are considered succulent euhalophytes. *Salicornia patula* Duval-Jouve is a frequent species in saline ecosystems on the Iberian Peninsula, on the coasts of France, Portugal, and Italy [2].

In terms of their elemental content, they accumulate inorganic salts, highlighting Na concentrations, with values higher than 20,000 mg/kg DW (dry weight) [3]. Significant values of K, Ca, and Mg are found in *S. patula* from Tinto river locations and other Mediterranean areas [4]. One of the bioactive compounds of interest in *Salicornia* is polyphenol, which in the field of nutrition has been of interest in recent decades. Polyphenols have been proven to have therapeutic effects for cardiovascular diseases, neurodegenerative disorders, cancer, and obesity [5].

The main classes of phenolic compounds are phenolic acids, flavonoids, stilbenes, and lignans. Flavonoids are present in plants as pigments that protect the body from damage caused by oxidizing agents [6]. In *Salicornia europaea* L. and *Salicornia herbacea* L. the presence of flavonoids such as quercetin, kaempferol, or catechin, among others, has been demonstrated [7].

Fatty acids are other bioactive compounds present in vegetables, and some of them, such as essential fatty acids, must be acquired through the diet since humans cannot synthesize them [6]. The presence of fatty acids in the *Salicornia* genus are found both in its stems and seeds, and some species such as *S. europaea* have been studied as a source of linoleic and oleic acids [6]. However, traditionally the consumption of *Salicornia* has been carried out through a mixture of different halophytes due to the taxonomic complexity involved in the Salicornioideae group [8]. This fact reduces functionality at the species and even genus level, where the diversity of bioactive compounds and their specificity in different groups of plants is of the utmost importance.

There is great interest in the study of phenolic compounds in “lesser-known” wild plants, since several studies have revealed the important role they played in human health [9]. Therefore, knowing the importance of new uses of *S. patula,* which has a long history of being gathered from the wild as a source of food [10], the objective of this work is to analyze for the first time the phenolic compounds and fatty acids in *S. patula*, both in the coastline marshes and inland salt marshes of the Iberian Peninsula.

## 2. Materials and Methods

Table 1 includes the data on the collection and geographical locations of *S. patula* selected samples from the Iberian Peninsula (Spain). Fleshy stems were selected until approximately 500 mg per sample was obtained for the analysis of phenolic compounds (phenolic acids and flavonoids) and fatty acids.

### 2.1. Preparation of the Methanol Extract

Five hundred milligrams of plant sample were selected with a solution of methanol in water (50 mL/50 mL) at ambient temperature, 25 °C. The extracts were filtered using a Whatman N° 4 filter. The solid residue was then extracted with 30 mL of methanol in water. The extracts were filtered again and re-dissolved with 10 mL of methanol in water. The extractions were performed in three replicates of each sample.

### 2.2. Total Phenolic Compounds (TPC)

**Figure 1:**
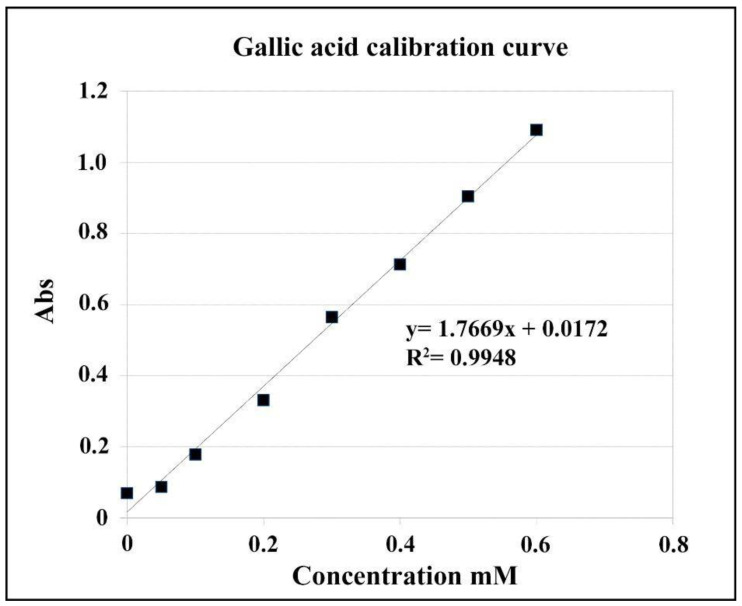
Gallic acid equivalent calibration curve.

Total phenol content was determined by the Folin–Ciocalteu method [11] using gallic acid (GA) as per the recommended standard [12]. From these solutions, an aliquot of 0.5 mL of methanolic extract was taken and 2.5 mL of the Folin–Ciocalteu reagent was added and it was left to react for 3 min, then 2 mL of Na_2_CO_3_ solution was added and mixed in a shaker (Heidolph, Berlin, Germany). The solution was incubated at a temperature of 40 °C in a dark stove for 1 h. The absorbance was measured at 765 nm using a spectrophotometer and the results were expressed in gallic acid equivalents, using a gallic acid (0.05–0.5 mg/mL) standard curve (Figure 1). To prepare a calibration curve 0.017 g of gallic acid was added, as control or standard, into 100 mL volumetric flasks, and then diluted to a volume of 100 mL with water. These solutions will have a known phenol concentration of 1 mM of gallic acid, the effective range of the assay (Table 2).

### 2.3. Liquid Chromatography–Mass Spectrometry (LC–MS)

Chromatographic separation was performed as follows: all of the supplied volume was first dried in a rotary evaporator (Rotavapor Fischer) and later in a lyophilizer Telstar. The total amount of sample obtained was weighed. They were derivatized with Meth-Prep Fisher (EEUU). Meth-Prep II is a 0.2 N methanolic solution of m-trifluoromethylphenyl trimethylammonium hydroxide. This one-step reagent simplifies the transesterification of triglycerides to methyl esters. They were injected into GC/MS.

The chromatographic separation was done in a HPLC-MS Agilent 6120 (Santa Clara, CA, USA) using a C20 column maintained at 35 °C. The chromatography mass spectrometry was carried out at the Servicio Interdepartamental de Investigación from the Universidad Autónoma de Madrid (UAM).

### 2.4. Electrospray Ionization Mass Spectometry (LC–ESI-MS/MS)

Flavonoids were determined using an HPLC-MS Agilent 1200 (Santa Clara, CA, USA) in a C20 column at 35 °C. The composition of the gradient solvent was as follows. The solvent system used was a gradient of acetonitrile (solvent A) and formic acid 2% (solvent B): 0 min, 4% of solvent A; 10 min, 10% of solvent A; 20 min, 20% of solvent A; 30 min, 40% of solvent A; 35 min, 40% of solvent A; 40 min, 60% of solvent A; 45 min, 60% of solvent A; and 55 min, 4% of solvent A. The flow rate was 1 mL/min and runs were monitored with a UV-visible photodiode array detector set at 280 nm (phenolic acids) and 360 nm (flavonols), for a total chromatogram time of 50 min. An injection volume of 5 µL was taken from a 1.2 mg/2 mL. This technique was used to identity the flavonoids in the extract according to their protonation [M + H] + (Table 2), to calculate the relative retention time of each peak in the chromatograms obtained by HPLC.

### 2.5. Statistical Analysis

Statistical analysis was performed in the Statgraphics 18.0 program. Mean and standard deviations were calculated. To test the possible differences between three or more groups, they were compared using an ANOVA analysis of variance for total phenolic compounds.

## 3. Results

### 3.1. Total Phenolic Compounds (TPC)

Samples with the highest content of phenolic compounds are from the La Rábida Tinto river (4.209 mg GA/g plant DW (dry weight)) (Table 3).

Samples from Aldemayor de San Matín (Valladolid) and Colmenar de Oreja (Madrid) show differences regarding their conservation, with 4.091 mg GA/g plant DW and 4.172 mg GA/g plant DW for dry material. For fresh material these samples contain 2.117 mg GA/g plant FW (fresh weight) and 1.313 mg GA/g plant FW.

The results of the statistical analysis are homogeneous and do not show significant differences between the mean values of the studied samples. Sample 6 is the only one that shows a higher standard deviation.

### 3.2. Phenolic Acids

**Figure 2:**
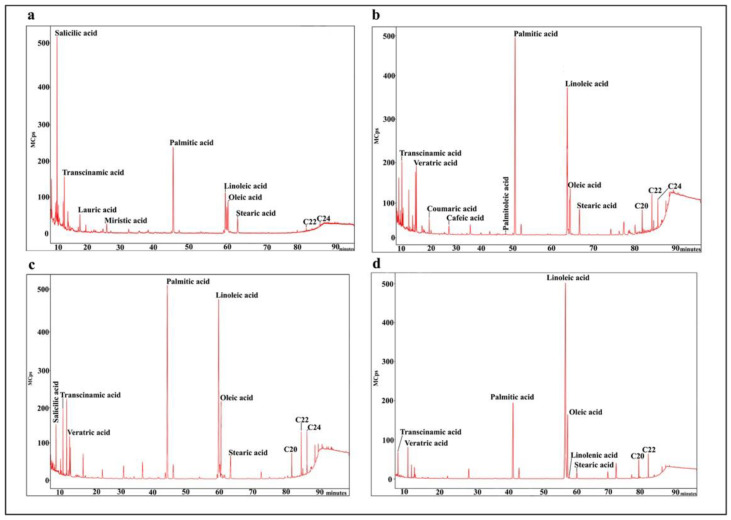
Chromatograms of the studied samples: phenolic acids and fatty acids. (**a**): Tinto river territories, samples 1–3, 6–9; (**b**): Odiel and Piedras river territories, samples 4–5; (**c**): mainland territories, samples 10–13; and (**d**): mainland territories, Valladolid, sample 11.

**Figure 3:**
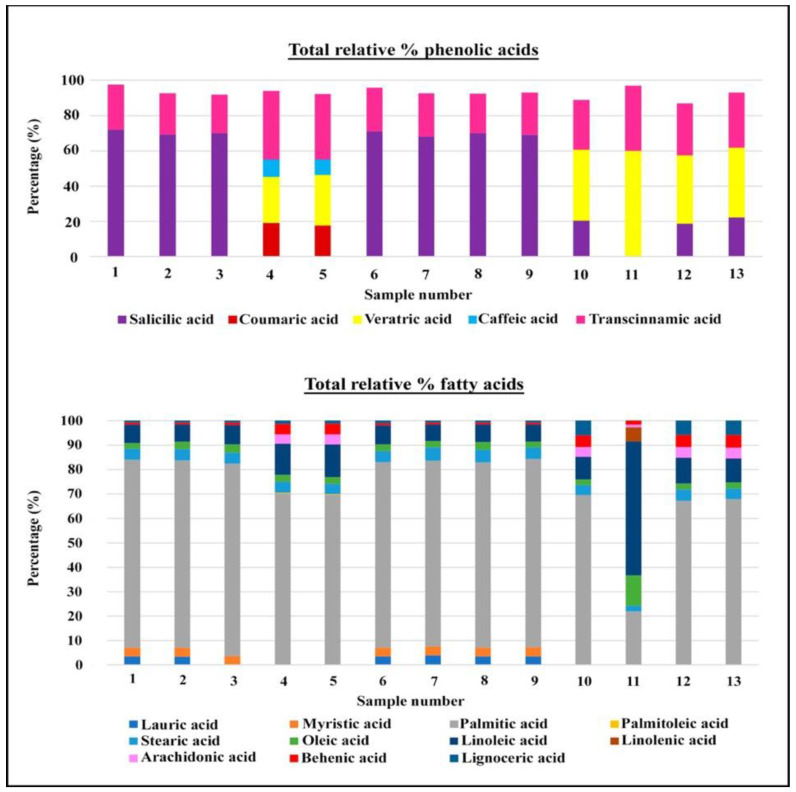
Total relative percentages of phenolic acids and fatty acids.

Transcinnamic acid is present in all *S. patula* samples. The materials from Tinto, Odiel, and Piedras rivers (Huelva) show relative transcinnamic acid contents between 21% and 39%. The samples collected in mainland territories from the Iberian Peninsula contain between 28% and 37% of the relative percentage (Figure 2 and Figure 3, and Table 4).

In the material from the Tinto river coastal saltmarsh in the Iberian southwest, salicylic acid stands out with more than 60% relative content. Samples from the mainland territories present no more than 22% (Figure 2 and Figure 3, and Table 4).

Samples 4 and 5 from Moguer and El Terrón also present veratric, coumaric, and caffeic acid with percentages close to 26%, 17%, and 8%, respectively (Figure 2).

Material from Aldeamayor de San Martín (samples 10 and 11) contains veratric acid with a relative content of 40–60%, and a transcinnamic acid relative content of 28–37%. Sample 10 also shows the presence of salicylic acid, with a relative content of 20%.

The material from Colmenar de Oreja (sample 13) contains veratric acid with a relative content of about 40%, transcinnamic acid with a relative content of more than 30%, and salicylic acid with a relative content of slightly more than 20% (Figure 2 and Figure 3, and Table 4).

### 3.3. Flavonoids

**Figure A1:**
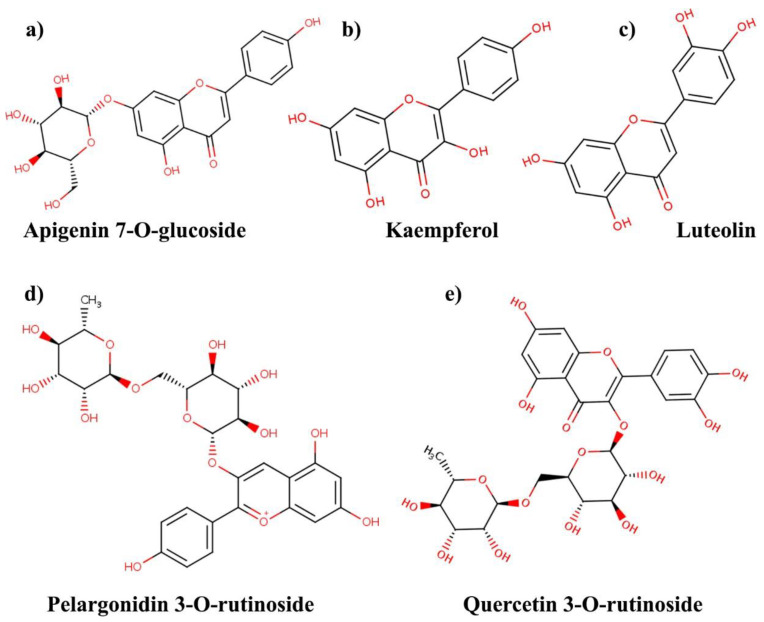
Structures of the identified flavonoids.

All samples of *S. patula* (Table 5) present quercetin-3-O-rutinoside or rutin, and apigenin 7-glucoside. Samples 4, 5, 10, 12, and 13 also present pelargonidin-3-O-rutinoside and luteolin/kaempferol (it has not been possible to determine with precision which of these two compounds are present since there is no standard and both have the same molecular weight (286.24 g/mol)) (Table 6). The chemical structures of these compounds were extracted from Phenol Explorer and are shown in Figure A1.

### 3.4. Fatty Acids

With respect to fatty acids, our results show that the proportion of total saturated fatty acids (SFAs), except for sample 11, account for 80% of the total 70%. Total monounsaturated fatty acids (MUFAs) account for around 2–3%, such as oleic acid and palmitoleic acid, with the exception of sample 11 from Valladolid where MUFAs account for 12%. Total polyunsaturated fatty acids (PUFAs) account for between 6% and 13%. Of these, linoleic is present in all of the samples but a higher concentration was reached in sample 11. Furthermore, this specimen from Aldeamayor de San Martín is the only one that contains linolenic acid (Figure 2 and Figure 3, and Table 7).

Plant materials from the Huelva marshes had differences between them. Samples 1, 2, 3, 6, 7, 8, and 9 contained lauric acid, myristic acid, and lignoceric acid (C24), whose relative content ranges between 0.5% and 3%; conversely, samples 4 and 5 contained arachidic acid (C20) and palmitoleic acid with a content between 0.18% and 4%.

The materials from Colmenar de Oreja (Madrid), samples 12 and 13, also present arachidic acid and lignoceric acid with a relative content of around 6% for both compounds.

## 4. Discussion

### 4.1. Phenolic Compounds

*S. patula* shows an increase in TPC values when the material is kept dry while the frozen or fresh material presents more modest values. The TPC data of dried *S. patula* range between 2.9 and 4.2 mg GA/g DW plant and are higher than those found in stems of *S. europaea* collected in Romania [13], with 1.04 mg G.A./g plant DW. Other authors describe in *S. europaea* higher values in stems samples from Turkey [14].

### 4.2. Phenolic Acids

The content of phenolic compounds in different halophytes has been related to their ability to survive in high salinity conditions. The wide diversity of different phenolic acids in *S. patula* may be understood as an adaptation mechanism to saline environments [15]. Among these, salicylic acid is present in the highest percentage in *S. patula*, with 70% relative content in the material collected in Huelva. It is the main phenolic acid in *S. europaea* [14,16]. Salicylic acid is involved with plant growth and acts as a genetic regulator against salt stress [17].

Transcinnamic acid is present in all of the studied samples of *S. patula* and ranges between 20% and 40% in content, standing out in interior provinces. Friedman and Jurgens [18] found that transcinnamic acid in plant samples can change depending on the pH of the soil. The differences in the pH values may explain the variation in the content of this acid in *S. patula* samples.

Veratric acid stands out in the material from inland provinces (Colmenar de Oreja and Aldeamayor de San Martín) with relative contents of up to 40%. Samples from Piedras and Odiel estuary rivers contain a lower percentage of this same bioactive compound. Veratric acid has antibacterial, anti-inflammatory, and antihypertensive activities [19].

Coumaric acid in *S. patula* has only been identified in the material collected in the Piedras and Odiel rivers, with a relative content of 19%. Our values for coumaric acid are higher than those published by Zengin et al. in *S. europaea* [14]. On the other hand, caffeic acid is the minor phenolic acid found in *S. patula*. In other *Salicornia* species, higher data have been described, such as in *S. europaea* from Turkey [14].

### 4.3. Flavonoids

Most flavonoids are present in plants as esters, glycosides, or polymers. The chemical structure of flavonoids determines the absorption range. For this reason, glycosylation guarantees that some flavonoids are absorbed, giving them prebiotic actions [20].

All the samples of *S. patula* studied have apigenin 7-glucoside and pelargonidin-3-O-rutinoside, both identified for the first time in *Salicornia* genus. Likewise, all samples have quercetin-3-O-rutinoside, commonly called rutin, an antioxidant compound that improves tolerance to salinity, already identified in other halophytes [14,21,22]

The presence of this flavonol in all *Salicornia* samples suggests it is an important adaptation to saline environments. Some of the *S. patula* samples (4, 5, 10, 12, and 13) also present luteolin or kaempferol. Luteolin has been previously identified in *S. europaea* [8].

### 4.4. Fatty Acids

Our results show that palmitic acid appears in the highest proportion in *S. patula* and can exceed 70%. In other *Salicornia* species, such as *Salicornia ramosissima* Woods this acid exceeded 20% [23], as in *S. europaea* [16], and *Salicornia bigelovii* Torr [24]. In other halophytes like *Arthrocnemum indicum is* also demonstrated the presence of this acid, with a relative content of more than 18% [25].

Among the monounsaturated fatty acids, palmitoleic acid is only present in the material collected from the southwest Iberian Peninsula. *S. patula* also contains oleic acid, another monounsaturated fatty acid, that has been described in *S. europaea* [16] and *S. bigelovii* [24]. These bioactive compounds prevent the development of cardiovascular disorders, reduce insulin resistance, and strengthen the immune system [8].

Polyunsaturated acids such as linolenic acid have been found in proportions of more than 5% in sample 11, as in other *Salicornia* species such as *S. bigelovii* [24]. In *S. ramosissima* the values for this fatty acid are lower than those found here [26]. Other authors in *Salicornia brachiate* Roxb reveal a content of 29% [26].

Linoleic acid showed a content of 56% in the samples collected in Aldeamayor de San Martín, Valladolid province. The total polyunsaturated acid content in this population is 61%, a value similar to that described by Patel et al. [27] for *S. brachiata*. Polyunsaturated fatty acids are bioactive compounds with antifungal activity, and additionally they inhibit carcinogenesis and the progression of atherosclerosis [28].

Long-chain fatty acids, such as arachidonic acid, behenic acid, and lignoceric acid, do not exceed a content of 6% in *S. patula*. El-Araby et al. [24] provide similar values in *S. bigelovii*. In other *Salicornia* species, long-chain fatty acids have been described to appear in lower values, such as *S. ramosissima* [22].

For all of this, the study of the evaluation of the antioxidant activity of *S. patula* extracts, through the appropriate tests, 2,2-diphenyl-1-picrylhydrazyl (DPPH) the 2,2′-azino-bis (3-ethylbenzothiazoline-6-sulphonic acid (ABTS) and the ferric reducing antioxidant power (FRAP), is necessary to complete the information on bioactive compounds and the differences between species of various origins. We are approaching the study of antioxidant activity in the *Salicornia* genus in the near future.

## 5. Conclusions

For the first time, the bioactive compounds (phenolic compounds and fatty acids) present in *S. patula* are described. The phenolic acids identified in all samples are salicylic, transcinnamic, and veratric acid. Samples from the coastal salt marshes and the Odiel, and Piedras rivers also presented caffeic and coumaric acid. The composition of phenolic acids and flavonoids, like rutin, may be related to adaptation mechanisms in saline environments. Other flavonoids detected for the first time in this species are apigenin-7-glucoside and pelargonidin 3-O-rutinoside, with glycosylated structures that confer their prebiotic properties.

The lipid profile shows the presence of palmitic, stearic, oleic, and linoleic as the main fatty acids. Lauric, myristic, and palmitoleic fatty acids were only detected in the material from the coastal salt marshes in lower proportions.

Finally, we show that *S. patula* is a source of bioactive compounds with important positive biological effects. Its consumption both in a traditional way, or as an additional ingredient, make this Mediterranean euhalophyte a functional food.

## Figures and Tables

**Table 1:** Information about the sampled localities for *S. patula*.

ID	Geographical Location and Collection Date	Latitude/Longitude
**1**	Spain, Huelva, La Rábida, Tinto river, 24 October 2017	37.21295617/−6.93198742
**2**	Spain, Huelva, La Rábida, Tinto river, 24 October 2017	37.21295617/−6.93198742
**3**	Spain, Huelva, La Rábida, Tinto river, 24 October 2017	37.21295617/−6.93198742
**4**	Spain, Huelva, Moguer, Odiel river, 02 December 2009	37.29613488/−7.05380284
**5**	Spain, Huelva, El Terrón, Piedras river, 19 September 2007	37.19231363/−7.33811922
**6**	Spain, Huelva, San Juan del Puerto, Tinto river, 11 May 2003	37.30121786/−6.82803804
**7**	Spain, Huelva, Tinto river estuary, 15 December 2017	37.21315236/−6.9432506
**8**	Spain, Huelva, Tinto river estuary, 15 December 2017	37.21315236/−6.9432506
**9**	Spain, Huelva, Tinto river estuary, 15 December 2017	37.21315236/−6.9432506
**10**	Spain, Valladolid, Aldeamayor de San Martín, 22 October 2017	41.50816286/−4.65960225
**11**	Spain, Valladolid, Aldeamayor de San Martín, 22 October 2017	41.50816286/−4.65960225
**12**	Spain, Madrid, Colmenar de Oreja, 10 November 2017	40.1379148/−3.5709978
**13**	Spain, Madrid, Colmenar de Oreja, 10 November 2017	40.1379148/−3.5709978

**Table 2:** Gallic acid data with absorption spectrophotometric values (*n* = 3).

Gallic Acid Curve	Concentration (mM)	Concentration (mg/mL)	ABS	ABS	ABS
Control	0	0.00	0.000	0.000	0.000
1	0.05	0.01	0.085	0.087	0.089
2	0.1	0.02	0.178	0.178	0.176
3	0.2	0.03	0.331	0.331	0.329
4	0.3	0.05	0.564	0.566	0.578
5	0.4	0.07	0.713	0.703	0.709
6	0.5	0.09	0.904	0.911	0.907
7	0.6	0.10	1.091	1.082	1.094

**Table 3:** Ratio of total phenolic compounds (TPC) determined by the Folin–Ciocalteu method. SD: standard deviation. For each column different superscript letters (a and b) mean statistically significant variations among samples.

ID	Sample Weight	TPC	SD	Material Conservation
**1**	0.581 g	3.128 mg GA/g plant ^a^	0.123	Fresh material
**2**	0.590 g	4.209 mg GA/g plant ^a^	0.154	Dry material
**3**	0.540 g	4.086 mg GA/g plant ^a^	0.027	Frozen material
**4**	0.524 g	3.171 mg GA/g plant ^a^	0.173	Dry material
**5**	0.565 g	3.143 mg GA/g plant ^a^	0.078	Dry material
**6**	0.531 g	3.428 mg GA/g plant ^b^	1.270	Dry material
**7**	0.581 g	1.372 mg GA/g plant ^a^	0.020	Fresh material
**8**	0.572 g	2.989 mg GA/g plant ^a^	0.893	Dry material
**9**	0.581 g	2.530 mg GA/g plant ^a^	0.118	Frozen material
**10**	0.584 g	4.091 mg GA/g plant ^a^	0.124	Dry material
**11**	0.595 g	2.117 mg GA/g plant ^a^	0.027	Fresh material
**12**	0.514 g	1.313 mg GA/g plant ^a^	0.011	Fresh material
**13**	0.591 g	4.172 mg GA/g plant ^a^	0.122	Dry material

**Table 4:** Relative content of phenolic compounds in *S. patula* samples. nd: not detected.

ID	Coumaric Acid	Salycilic Acid	Veratric Acid	Caffeic Acid	Transcinnamic Acid
**1**	nd	72.04 (0.02)	nd	nd	25.60 (0.05)
**2**	nd	69.30 (0.01)	nd	nd	23.45 (0.04)
**3**	nd	70.08 (0.01)	nd	nd	21.90 (0.02)
**4**	19.07 (0.06)	nd	26.06 (0.02)	9.88 (0.02)	39.08 (0.11)
**5**	17.76 (0.05)	nd	28.66 (0.01)	8.70 (0.00)	37.12 (0.01)
**6**	nd	71.08 (0.04)	nd	nd	24.68 (0.01)
**7**	nd	68.12 (0.11)	nd	nd	24.50 (0.09)
**8**	nd	70.22 (0.05)	nd	nd	22.21 (0.04)
**9**	nd	69.04 (0.06)	nd	nd	24.02 (0.03)
**10**	nd	20.46 (0.01)	40.16 (0.02)	nd	28.33 (0.03)
**11**	nd	nd	59.98 (0.02)	nd	37.12 (0.01)
**12**	nd	8.69 (0.02)	38.77 (0.01)	nd	29.50 (0.05)
**13**	nd	22.31 (0.01)	39.44 (0.02)	nd	31.41 (0.01)

**Table 5:** Tentative identification flavonoids in *S. patula.*

Flavonoid Compound	Experimental Mass M-H m/z	MS/MS (m/z)
Quercetin-3-O-rutinoside (rutin)	610	609–610
Apigenin-7-glucoside	433	433
Kaempferol/Luteolin	287	285–286
Pelargonidin-3-O-rutinoside	580	577–579

**Table 6:** Main tentative flavonoids in *S. patula* according to their retention time. Numbers 1–13 correspond to the identification of the studied materials collected in Table 1. +++ = presence.

Retention Time (min)	Tentative Identity of Flavonoids	1	2	3	4	5	6	7	8	9	10	11	12	13
16	Luteolin/Kaempferol				+++	+++					+++		+++	+++
17.6	Luteolin/Kaempferol				+++	+++					+++		+++	+++
19	Luteolin/Kaempferol				+++	+++					+++		+++	+++
21.3	Apigenin-7-glucoside	+++	+++	+++	+++	+++	+++	+++	+++	+++		+++		
21.9	Pelargonidin-3-O-rutinoside				+++	+++					+++		+++	+++
23.6	Luteolin/Kaempferol				+++	+++					+++		+++	+++
25.5	Rutin	+++	+++	+++	+++	+++	+++	+++	+++	+++	+++	+++	+++	+++
26	Luteolin/kaempferol				+++	+++					+++		+++	+++
27.6	Apigenin-7-glucoside	+++	+++	+++	+++	+++	+++	+++	+++	+++	+++	+++	+++	+++
27.8	Pelargonidin-3-O-rutinoside				+++	+++					+++		+++	+++
28	Rutin	+++	+++	+++	+++	+++	+++	+++	+++	+++	+++	+++	+++	+++
28.8	Luteolin/Kaempferol				+++	+++					+++		+++	+++
29.1	Luteolin/Kaempferol				+++	+++					+++		+++	+++
29.5	Luteolin/Kaempferol				+++	+++					+++		+++	+++
31.1	Pelargonidin-3-O-rutinoside				+++	+++					+++		+++	+++
31.4	Rutin	+++	+++	+++	+++	+++	+++	+++	+++	+++	+++	+++	+++	+++
37.4	Apigenin-7-glucoside	+++	+++	+++	+++	+++	+++	+++	+++	+++	+++	+++	+++	+++
39.5	Luteolin/Kaempferol				+++	+++					+++		+++	+++
42.5	Apigenin-7-glucoside	+++	+++	+++	+++	+++	+++	+++	+++	+++	+++	+++	+++	+++
44.8	Pelargonidin 3-O-rutinoside				+++	+++					+++		+++	+++
47.8	Pelargonidin-3-O-rutinoside				+++	+++					+++		+++	+++

**Table 7:** Relative content of fatty acids (%) in *S. patula* by HPLC-values expressed as: mean (standard deviation, *n* − 1) *n* = 3. Legend: Lauric Acid (C12:0); Myristic acid (C14:0); Palmitic acid (C16:0); Palmitoleic acid (C16:1); Stearic acid (C18:0); Oleic acid (C18:1); Linoleic acid (C18:2); Linolenic acid (C18:3); Arachidonic acid (C20:0); Behenic acid (C22:0); Lignoceric acid (C24:0); SFAs (saturated fatty acids); MUFAs (monounsaturated fatty acids); PUFAs (polyunsaturated fatty acids). nd: not detected.

ID	Lauric Acid (C12:0)	Myristic Acid (C14:0)	Palmitic Acid (C16:0)	Palmitoleic Acid (C16:1)	Stearic Acid (C18:0)	Oleic Acid (C18:1)	Linoleic Acid (C18:2)	Linolenic Acid (C18:3)	Arachidonic Acid (C20:0)	Behenic Acid (C22:0)	Lignoceric Acid (C24:0)	TOTAL SFAs	TOTAL MUFAs	TOTA PUFAs
**1**	3.22 (0.05)	3.35 (0.07)	70.45 (0.62)	nd	4.12 (0.07)	2.17 (0.02)	6.88 (0.02)	nd	nd	0.77 (0.01)	0.71 (0.00)	82.82	2.17	6.88
**2**	3.28 (0.06)	3.42 (0.08)	72.96 (0.77)	nd	4.44 (0.11)	2.82 (0.01)	6.75 (0.04)	nd	nd	0.75 (0.01)	0.69 (0.02)	85.54	2.82	6.75
**3**	nd	3.31 (0.09)	69.66 (0.31)	nd	4.05 (0.06)	3.09 (0.09)	7.02 (0.07)	nd	nd	0.78 (0.01)	0.72 (0.01)	78.52	3.09	2.02
**4**	nd	nd	71.20 (0.28)	0.17 (0.00)	4.61 (0.12)	2.88 (0.23)	12.90 (0.01)	nd	3.92 (0.01)	4.31 (0.11)	1.35 (0.01)	85.39	3.05	12.90
**5**	nd	nd	69.30 (0.33)	0.18 (0.01)	4.28 (0.03)	2.66 (0.12)	13.33 (0.04)	nd	4.08(0.02)	4.38 (0.05)	1.21 (0.00)	83.25	2.84	13.33
**6**	3.25 (0.01)	3.33 (0.11)	70.43 (0.55)	nd	4.23 (0.09)	2.55 (0.05)	7.11 (0.05)	nd	nd	0.81 (0.02)	0.98 (0.01)	83.03	2.55	7.11
**7**	3.72 (0.00)	3.41 (0.09)	71.08 (0.26)	nd	5.02 (0.08)	2.42 (0.07)	6.42 (0.04)	nd	nd	0.73 (0.02)	0.66 (0.01)	84.60	2.42	6.42
**8**	3.31 (0.02)	3.37 (0.02)	71.41 (0.32)	nd	4.87 (0.09)	3.01 (0.11)	6.77 (0.02)	nd	nd	0.73 (0.01)	0.66 (0.01)	84.35	3.01	6.77
**9**	3.33 (0.07)	3.44 (0.06)	72.50 (0.22)	nd	4.56 (0.04)	2.12 (0.05)	6.70 (0.01)	nd	nd	0.71 (0.01)	0.70 (0.02)	85.23	2.12	6.70
**10**	nd	nd	71.83 (0.04)	nd	4.24 (0.06)	2.49 (0.01)	9.55 (0.02)	nd	4.14 (0.01)	5.00 (0.07)	6.11 (0.03)	87.09	2.49	9.55
**11**	nd	nd	21.51 (0.81)	nd	2.35 (0.01)	12.81 (0.03)	56.02 (0.04)	5.88 (0.07)	1.08 (0.06)	1.69 (0.10)	nd	26.63	12.81	61.90
**12**	nd	nd	69.80(0.35)	nd	4.71 (0.08)	2.63 (0.04)	10.88 (0.05)	nd	4.52 (0.03)	5.19 (0.01)	6.04 (0.02)	84.29	2.63	10.88
**13**	nd	nd	70.57 (0.51)	nd	4.56 (0.07)	2.56 (0.08)	10.31 (0.02)	nd	4.61 (0.02)	5.31 (0.03)	6.12 (0.07)	86.17	2.56	10.31

## Data Availability

Data is contained within the article or supplementary material.
